# Pharmacological intervention of Chinese medicine via regulating ferroptosis in gastric cancer

**DOI:** 10.1186/s13020-025-01314-8

**Published:** 2026-01-12

**Authors:** Hongchen Zhang, Xiaoxue Du, Jian Chen, Linhao Xu

**Affiliations:** 1 Department of Gastroenterology, Affiliated Hangzhou First People’s Hospital, School of Medicine, Westlake University, Hangzhou, 310006 Zhejiang China; 2 Translational Medicine Research Center, Key Laboratory of Clinical Cancer Pharmacology and Toxicology Research of Zhejiang Province, Affiliated Hangzhou First People’s Hospital, School of Medicine, Westlake University, Hangzhou, 310006 Zhejiang China; 3 School of Basic Medical Sciences and Forensic Medicine, Hangzhou Medical College, No. 481 Binwen Road, Binjiang District, Hangzhou, 310053 Zhejiang China; 4 Department of Cardiology, Affiliated Hangzhou First People’s Hospital, School of Medicine, Westlake University, #261 Huansha Road, Shangcheng District, Hangzhou, 310006 Zhejiang China

**Keywords:** Gastric cancer, Ferroptosis, Traditional Chinese medicine, Drug resistance

## Abstract

Gastric cancer (GC) is the second most frequently diagnosed malignancy, as well as the second most common cause of cancer-related deaths in China. Drug resistance is a major factor that limits the efficacy of GC chemotherapy. Given the increased resistance of GC cells to ferroptosis, activating the ferroptotic pathways has emerged as a promising therapeutic strategy against GC. This review summarizes the pathways involved in ferroptosis resistance in GC cells and the mechanisms underlying the therapeutic effects of herbal formulae and their bioactive compounds, with particular emphasis on ferroptosis. Multiple signaling pathways are implicated in regulating ferroptosis in GC cells, including the Wnt/β-catenin, PI3K/AKT/mTOR, TGF-β1/Smad, NF-κB, and Hippo pathways. According to previous clinical trials, traditional Chinese medicine (TCM) formulations can prolong the survival or increase survival chances in patients with GC, and reduce adverse reactions, thereby improving the quality of life. Finally, preclinical studies have shown TCMs and their bioactive compounds can ameliorate GC progression by triggering ferroptosis. Despite these beneficial effects on patients with GC, the underlying molecular mechanisms of TCM in GC have not been fully elucidated yet, and there are also some crucial shortcomings in the current studies. Therefore, further clinical trials and experimental studies are required to deepen our understanding of the mechanisms for activating ferroptosis in GC cells through TCM.

**Graphical Abstract:**

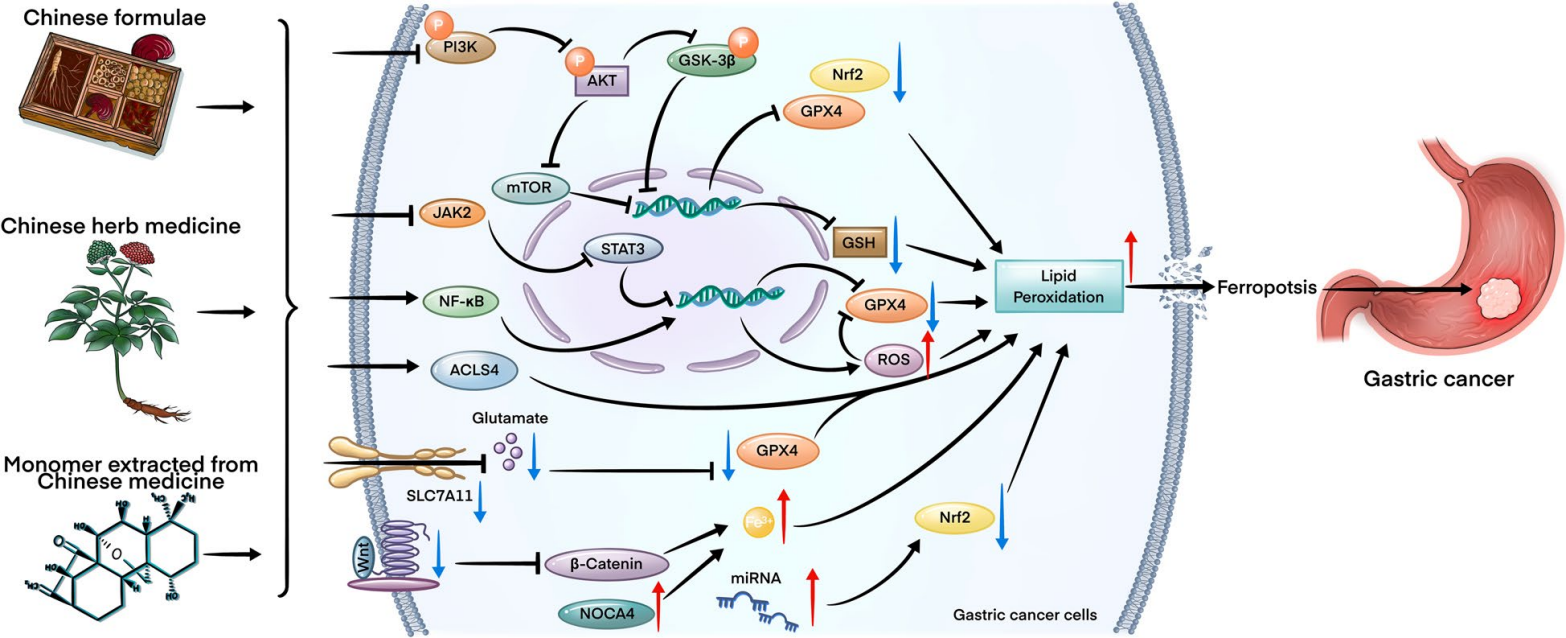

## Introduction

Gastric cancer (GC) is the second most frequently diagnosed malignancy and the second most common cause of cancer-related deaths in China [1]. Over one million new cases of GC were diagnosed in 2020 alone, and almost half of the new cases and deaths occurred in China each year [2], resulting in a considerable economic burden. Recent advances in surgical techniques, radiotherapy, chemotherapy and neoadjuvant therapy have significantly improved the 5-year survival rates of patients with GC. However, GC cells often develop resistance to chemotherapy and targeted therapies, which decreases therapeutic efficacy. Moreover, the significant side effects associated with chemotherapeutic drugs severely affect the patients’ quality of life.

Ferroptosis is an iron-dependent form of cell death characterized by excessive lipid peroxidation. Unlike apoptosis, necrosis, or autophagy, ferroptosis is triggered by abnormally high iron levels in cells, which result in the accumulation of lipid peroxides [3]. GC cells are typically resistant to ferroptosis due to increased expression of ferritin [4] and glutathione peroxidase 4 (GPX4) [5], which promotes tumor growth and contributes to chemoresistance. Given the widespread resistance to apoptosis in GC cells, induction of ferroptosis pathways has emerged as a potential strategy for treating GC [6].

Traditional Chinese medicine (TCM) formulations have demonstrated clinical efficacy against GC and other cancers, which can be attributed to their numerous active ingredients that act on multiple targets and synergistically regulate different pathological processes in the tumor cells [7]. Furthermore, these formulations have relatively fewer side effects. In addition to relieving cancer symptoms and improving quality of life, they can also reduce the pain associated with chemotherapy or radiotherapy [8]. Although no clinical trials have demonstrated TCM-induced ferroptosis in patients with GC, there is pre-clinical evidence that some TCMs can attenuate the progression of GC by inducing ferroptosis.

This review summarizes the molecular mechanisms underlying the resistance of GC cells to ferroptosis and explores the putative mechanisms through which herbal medicines target ferroptosis and inhibit GC progression.

## Ferroptosis and its function in GC

The dysregulation of iron homeostasis represents an important molecular mechanism underlying ferroptosis induction. Under normal physiological conditions, the systemic iron transport occurs through transferrin-mediated circulation of ferric iron (Fe^3+^), which is then internalized into the cells via transferrin receptor 1 (TFR1)-dependent endocytosis. The intracellular Fe^3+^ is subsequently reduced to ferrous iron (Fe^2+^) by six-transmembrane epithelial antigen of prostate 3 (STEAP3), followed by cytosolic transport through divalent metal transporter 1 (DMT1) or extracellular export via ferroportin (FPN) [9]. The labile iron pool is tightly regulated through ferritin-mediated storage mechanisms. Notably, in GC, there are adaptive alterations in iron regulatory pathways to evade ferroptosis, such as downregulating a critical mediator of ferroptosis, nuclear receptor coactivator 4 (NCOA4), thereby diminishing reactive iron accumulation [10]. Although TFR1 overexpression in GC enhances cellular iron acquisition, establishing an “iron-addicted” phenotype[11], the high expression of DMT1 and FPN might decrease the iron accumulation in the cytoplasm.

Iron overload increases membrane lipid peroxidation through the Fenton reaction, wherein redox-active Fe^2+^ catalyzes peroxide decomposition into hydroxyl radicals. These reactive oxygen species (ROS) initiate oxidation of polyunsaturated fatty acid (PUFA)-containing phospholipids, thereby generating lipid hydroperoxides (PUFA-PL-OOH) via enzymatic oxidation by arachidonate lipoxygenases (ALOXs), lipoxygenase (LOX) isoforms, and cytochrome P450 oxidoreductase (POR) [12]. The cytotoxic accumulation of lipid peroxidation products, including malondialdehyde (MDA) and 4-hydroxynonenal (4-HNE), induces destabilization of the plasma membrane and catastrophic cell rupture [13]. Clinical evidence reveals that ALOX15 suppression in GC could promote chemoresistance by reducing lipid ROS accumulation [14], thus highlighting the therapeutic implications of lipid peroxidation regulation.

The glutathione (GSH)-GPX4 axis constitutes the primary antioxidant defense against ferroptosis [15]. GPX4 catalyzes the reduction of PUFA-PL-OOH to non-reactive phospholipid alcohols (PUFA-PL-OH), thereby maintaining membrane redox homeostasis. GPX4 overexpression in GC is prognostically significant, correlating with advanced disease progression and therapeutic resistance [16], while pharmacologically enhancing GPX4 degradation can restore ferroptosis sensitivity [17]. The tripeptide GSH biosynthesis depends on cystine uptake through the cystine/glutamate antiporter (xCT), a heterodimer composed of solute carrier family 7 member 11 (SLC7A11) and solute carrier family 3 member 2 (SLC3A2) subunits. Patients with GC exhibit high elevated expression of both transporters [18, 19], suggesting a mechanistic basis for ferroptosis resistance through enhanced GSH synthesis and GPX4 stabilization.

**Fig. 1:**
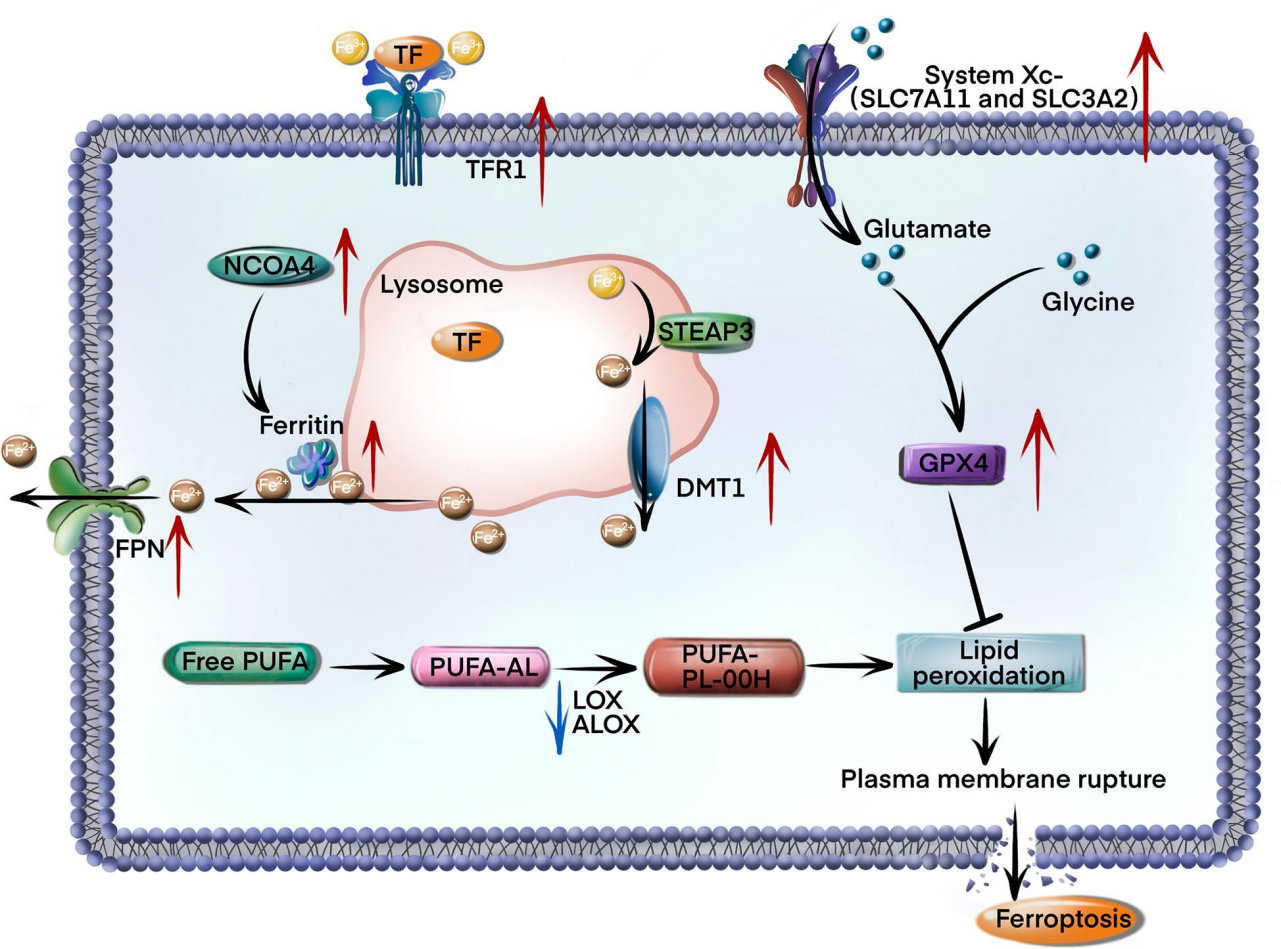
Schematic representation of the molecular mechanism of suppressing ferroptosis in GC. The expression levels of transferrin receptor 1 (TFR1), divalent metal transporter 1 (DMT1), ferroportin (FPN), solute carrier family 7 member 11 (SLC7A11) and glutathione peroxidase 4 (GPX4) were increased in GC tissue, while the expression of arachidonate lipoxygenases (ALOXs) was decreased

Emerging therapeutic strategies, such as targeting iron metabolism reprogramming, antioxidant capacity modulation, and lipid peroxidation potentiation, have demonstrated significant potential for overcoming ferroptosis resistance in GC. Combinatorial approaches addressing these interconnected pathways may yield novel precision therapies for GC (Fig. 1).

## Pathways regulating ferroptosis in GC

Multiple signaling pathways that are dysregulated in GC cells are associated with ferroptosis. These pathways and their regulatory mechanisms are summarized in this section.

### Wnt/β-catenin signaling pathway

**Fig. 2:**
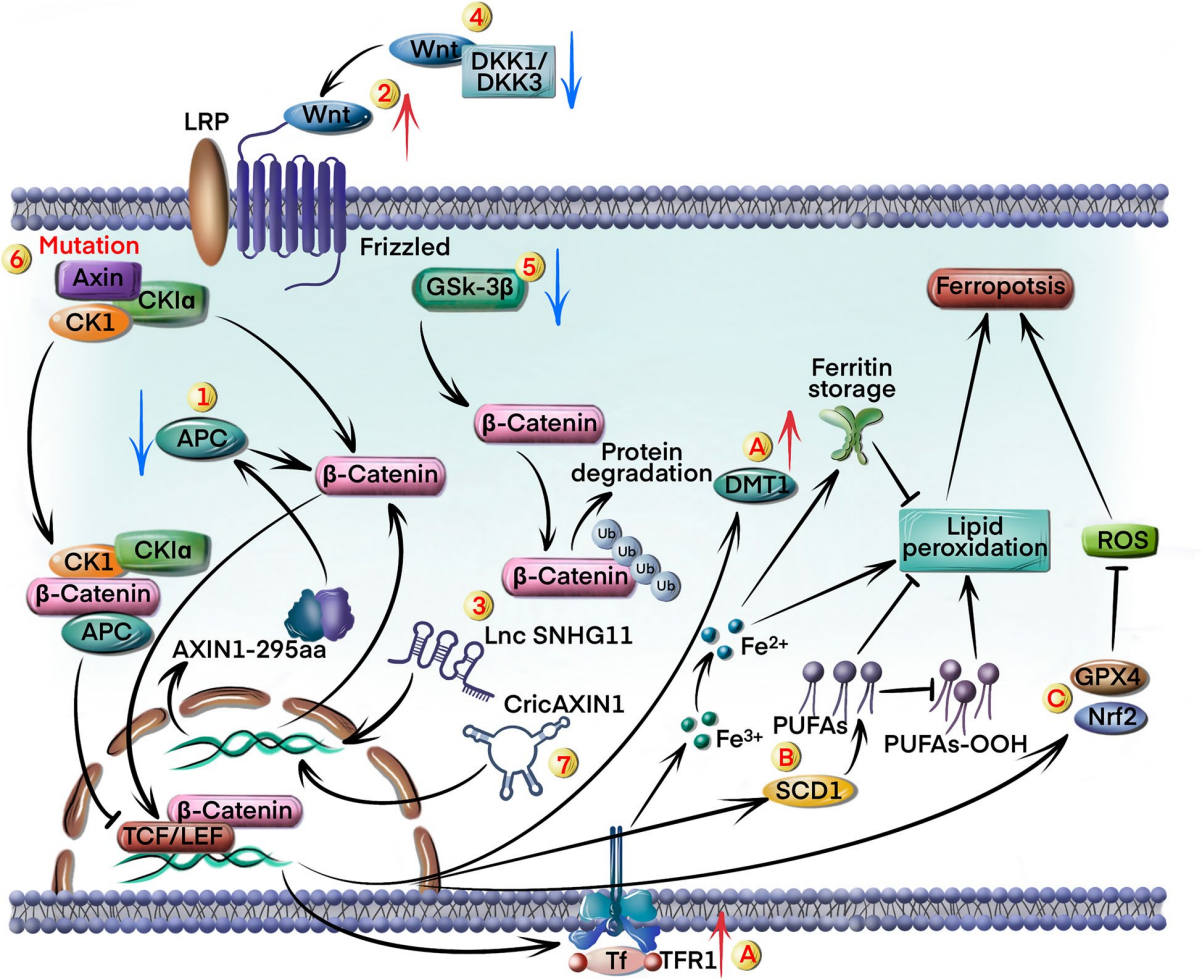
Activation of Wnt/β-catenin signaling pathway in GC cells inhibits ferroptosis. Multiple mechanisms contribute to the activation of Wnt/β-catenin signaling pathway: 1) mutations in the *APC* gene induce β-catenin to enter nucleus; 2) upregulation of Wnt ligands; 3) upregulation of SNHG11 inhibits β-catenin degradation; 4) downregulation of DKK1 and DKK3 facilitates Wnt ligands binding to Frizzled receptors; 5) reduction in GSK-3β activity leads to the accumulation of β-catenin; 6) mutations in the *Axin* gene lead to the accumulation of β-catenin; 7) upregulation of CircAXIN1 promotes nuclear translocation of β-catenin. The Wnt/β-catenin pathway inhibits ferroptosis through various mechanisms: A) upregulation of TFR1 and DMT1; B) accumulation of PUFAs through increased activity of SCD1 and PUFAs-OOH inhibition; C) activation of GPX4 and upregulation of Nrf2

The Wnt/β-catenin signaling pathway is a highly conserved pathway that regulates embryonic development, tissue homeostasis, cell proliferation, and cancer [20]. Numerous molecular mechanisms trigger Wnt/β-catenin activation in GC cells (Fig. 2).

A study showed that adenoma polyposis coli (APC), an inhibitory factor for the Wnt/β-catenin pathway, is highly expressed in GC cells [21]. APC forms a complex with Axin and glycogen synthase kinase-3β (GSK-3β), which promotes β-catenin degradation. Mutations or deletions in the *APC* gene can disrupt the function of this complex, resulting in the accumulation of β-catenin in the cytoplasm and nucleus, as well as the persistent activation of the Wnt/β-catenin pathway [22] (circle marked 1 in Fig. 2). Furthermore, the Wnt ligand Wnt1 is overexpressed in GC cells and continuously activates the downstream pathway [23] (circle marked 2 in Fig. 2). Catenin beta 1 (*CTNNB1*), the β-catenin encoding gene, is also upregulated in GC cells due to the increased expression of the lncRNA small nucleolar host gene 11 (*SNHG11*) [24]. Furthermore, GC cells exhibit mutations in CTNNB1 exon 3, which protect β-catenin from degradation. This leads to the accumulation of β-catenin and the ensuing activation of Wnt/β-catenin signaling [25] (circle marked 3 in Fig. 2). Dickkopf-related protein 1 (DKK1) and DKK3 suppresses the Wnt/β-catenin pathway by inhibiting the binding of Wnt ligands to Frizzled receptors [26] (circle marked 4 in Fig. 2). The Wnt inhibitors, including DKK1 [27], DKK4 [28], and secreted frizzled-related protein 4 (sFRP4) [29], are either downregulated or inactivated in GC cells, leading to aberrant Wnt/β-catenin activation. GSK-3β is a key negative regulator of Wnt/β-catenin signaling, and mediates the phosphorylation and degradation of β-catenin [30]. Reduced activity of GSK-3β in GC cells also leads to β-catenin accumulation [31] (circle marked 5 in Fig. 2). Axin mediates the formation of β-catenin degradation complexes, and mutations in the *Axin* gene could disrupt the stability of this complex, leading to the accumulation of β-catenin and abnormal activation of the downstream pathway [32] (circle marked 6 in Fig. 2). A recent study showed that CircAXIN1 could encode a 295 amino acid (aa)-long protein (AXIN1-295aa), which is overexpressed in GC tissues. AXIN1-295aa competitively interacts with APC and destabilizes the "destruction complex" of the Wnt pathway, resulting in the nuclear translocation of β-catenin [33] (circle marked 7 in Fig. 2). Thus, aberrant activation of the Wnt/β-catenin pathway in GC cells results from multiple synergistic mechanisms.

The Wnt/β-catenin pathway regulates multiple downstream effectors and target genes, including ferroptosis-associated genes. The molecular mechanisms underlying the regulation of ferroptosis by this pathway are also complex. Wnt/β-catenin signaling might increase the expression of iron transport-related proteins such as TFR1 and DMT1, thereby impairing iron uptake and distribution and reducing susceptibility to iron-mediated death [34, 35] (circle marked A in Fig. 2). In addition, the Wnt/β-catenin pathway plays an important role in lipid metabolism, especially in the synthesis and metabolism of PUFAs. Stearoyl-CoA desaturase 1 (SCD1), a downstream target of Wnt/β-catenin, converts saturated fatty acids into monounsaturated fatty acids and increases the accumulation of PUFAs to attenuate ferroptosis [36] (circle marked B in Fig. 2). Wnt/β-catenin signaling also inhibits ferroptosis indirectly by enhancing the cellular antioxidant mechanisms. Inhibition of this pathway sensitizes GC cells to ferroptosis by suppressing GPX4 activity [37]. Furthermore, Wnt/β-catenin activation induces transcription of various antioxidant genes via nuclear factor-E2-related factor 2 (Nrf2), which could prevent oxidative stress-induced ferroptosis [38] (circle marked C in Fig. 2).

In conclusion, the hyperactivation of the Wnt/β-catenin pathway inhibits ferroptosis in GC by targeting GPX4 and Nrf2. Pharmacological inhibition of Wnt/β-catenin could strategically sensitize GC cells to ferroptosis induction, offering a promising synergistic approach to overcome treatment resistance and improve patient outcomes.

### PI3K/AKT/mTOR signaling pathway

**Fig. 3:**
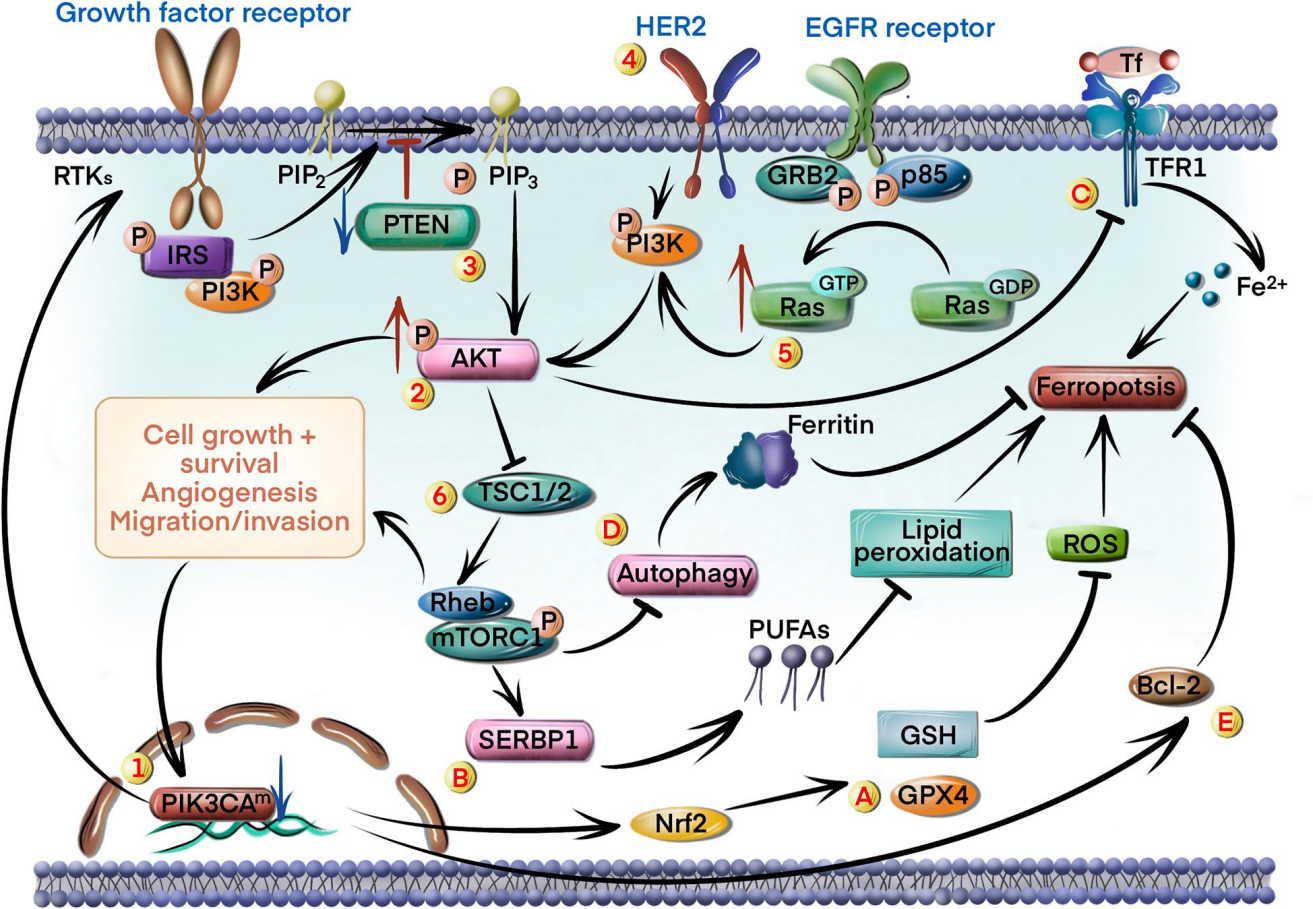
The PI3K/AKT/mTOR signaling pathway suppresses ferroptosis in GC cells. Several mechanisms activating the PI3K/AKT/mTOR pathway include: 1) mutations in the PIK3CA gene inducing PI3K/AKT activation e; 2) AKT hyperactivation; 3) mutations and deletions in the PTEN gene promoting PIP3 and AKT phosphorylation; 4) overexpression of HER2 promoting PIP3 and AKT phosphorylation; 5) activation of Ras; 6) inactivation of TSC1 and TSC2. The PI3K/AKT/mTOR pathway protects cells against ferroptosis through different mechanisms: A) upregulation of GPX4 and GSH via activating Nrf2; B) increasing the proportion of PUFAs via SREBP1 activation; C) downregulation of the cell surface transferrin receptor; D) autophagy inhibition through mTORC1; E) upregulation of anti-apoptotic proteins

Several mutations have been identified that induce persistent activation of the PI3K/AKT/mTOR signaling pathway. For instance, patients with GC often harbor mutations in the gene encoding phosphatidylinositol-4,5-bisphosphate 3-kinase alpha subunit (PIK3CA) [39], which have been linked to PI3K/AKT activation and GC development [40] (circle marked 1 in Fig. 3). Furthermore, the *AKT* gene is frequently overexpressed in GC cells [41], leading to sustained activation of the PI3K/AKT pathway and downstream mTOR signaling (circle marked 2 in Fig. 3).

Phosphatase and tensin homolog (PTEN) is a tumor suppressor protein that inhibits PI3K/AKT signaling by dephosphorylating PIP3 [42]. Mutations and deletions in the *PTEN* gene are associated with abnormal activation of the PI3K/AKT signaling pathway in GC tissues [43, 44] (circle marked 3 in Fig. 3). Human epidermal growth factor receptor-2 (HER2), a member of the epidermal growth factor receptor (EGFR) family, is commonly overexpressed in GC [45]. HER2 activation can promote PI3K/AKT signaling through dimerization and autophosphorylation, resulting in increased growth, proliferation, and survival of tumor cells [46, 47] (circle marked 4 in Fig. 3).

Ras protein also activates the downstream PI3K/AKT pathway by directly interacting with PI3K. Under normal physiological conditions, RAS switches between the GDP-bound inactive state and the GTP-bound active state. In GC cells, mutations in the GTP binding domain lead to sustained activation of RAS [48] and downstream PI3K/AKT/mTOR pathway, thereby promoting tumor growth and resistance to apoptosis [40] (circle marked 5 in Fig. 3). Tuberous sclerosis complex (TSC1-TSC2) is a negative regulator of RAS homolog enriched in brain (Rheb), which activates the mechanistic target of rapamycin complex 1 (mTORC1) [49]. The inactivation of the *TSC1* and *TSC2* genes in GC cells has been linked to overactivation of the mTOR signaling pathway [50] (circle marked 6 in Fig. 3).

The PI3K/AKT/mTOR pathway affects the susceptibility to ferroptosis by regulating cellular metabolism, antioxidant defense, and iron homeostasis. GPX4 is a key inhibitor of ferroptosis and protects cell membranes from oxidative damage by reducing lipid peroxides [51]. Activation of AKT could upregulate glutathione (GSH) and GPX4 in GC cells via Nrf2, thereby inhibiting ferroptosis [52] (circle marked A in Fig. 3). Additionally, the PI3K/AKT/mTOR pathway enhances antioxidant defense by promoting the synthesis of GSH, thereby inhibiting ferroptosis [53] (circle marked A in Fig. 3). Furthermore, mTORC1 can upregulate fatty acid synthase by activating sterol regulatory element binding protein 1 (SREBP1) [54], which increases the proportion of PUFAs in the cell membrane and reduces the abundance of lipid peroxidation substrates required for ferroptosis [55] (circle marked B in Fig. 3). AKT also decreases cell surface TFR-mediated iron uptake and promotes FPN-mediated iron transport, thereby altering free iron levels in the cells [56] (circle marked C in Fig. 3). Given that intracellular iron accumulation triggers iron-mediated cell death, AKT activation can inhibit ferroptosis. The mTOR protein is an important inhibitor of autophagy, a self-catabolic process for clearing damaged organelles. Autophagy inhibition following mTORC1 activation could suppress ferroptosis by reducing the degradation of ferritin [57], thereby protecting cells from iron-mediated death [58] (circle marked D in Fig. 3). The PI3K/AKT pathway also enhances cell survival by inhibiting pro-apoptotic proteins, such as Bcl-2-associated death promoter (BAD) and caspase, and upregulating anti-apoptotic proteins, such as Bcl-2 [59]; this indirectly affects sensitivity to ferroptosis (circle marked E in Fig. 3).

As discussed above, in addition to upregulating GSH and GPX4, the PI3K/AKT/mTOR pathway also increases the activity of fatty acid synthase, promotes FPN-mediated iron transport, and reduces the degradation of ferritin to suppress ferroptosis. Therefore, aberrant activation of the PI3K/AKT/mTOR pathway inhibits ferroptosis through multiple effectors and warrants further investigation as a potential target for cancer treatment strategies involving ferroptosis induction.

### TGF-β1/Smad signaling pathway

**Fig. 4:**
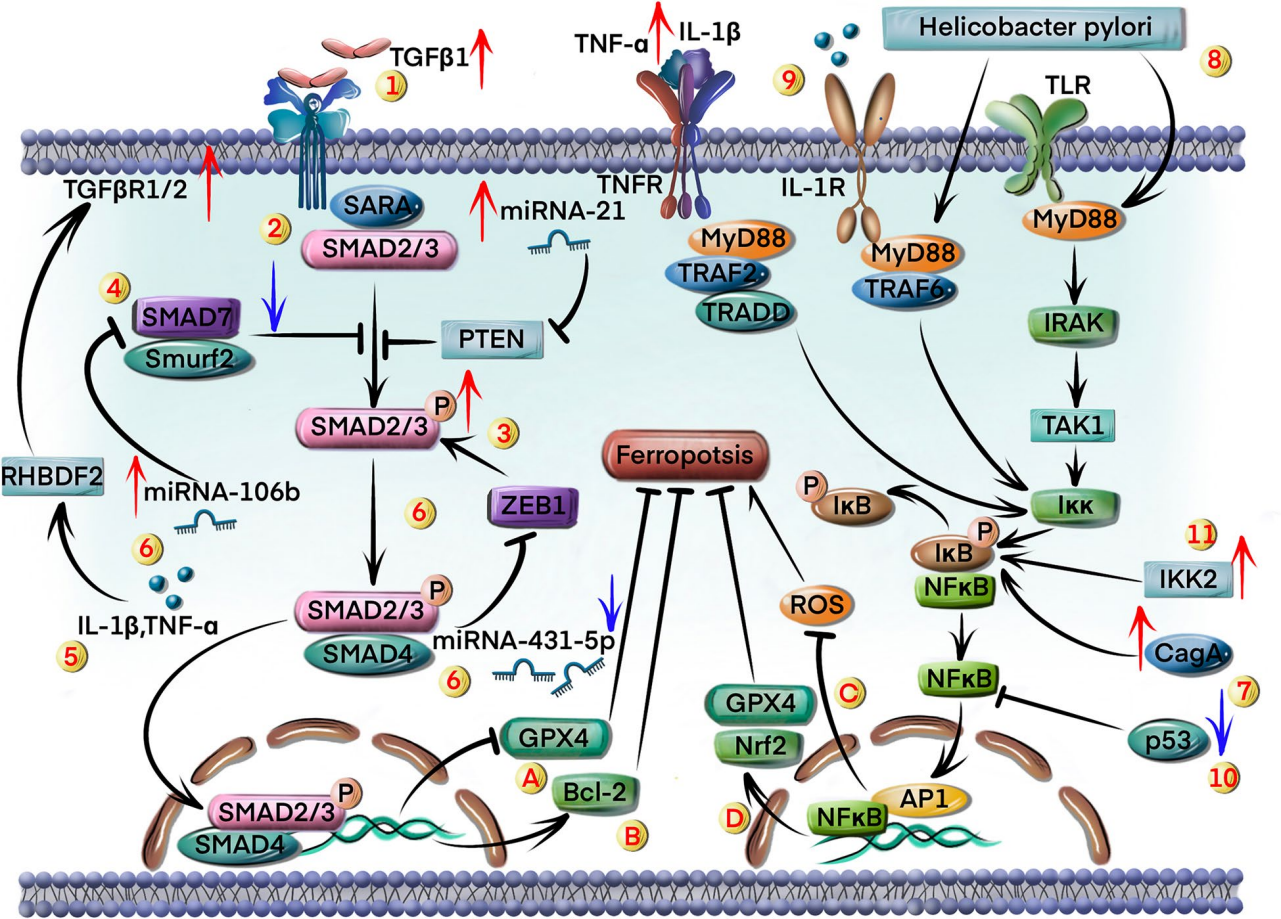
The TGF-β1/Smad and NF-κB pathways regulate ferroptosis in GC. TGF-β1/Smad signaling pathway is activated through various mechanisms: 1) overexpression of TGF-β1; 2) overexpression of TGF-β1 type 1 and 2 receptor; 3) excessive phosphorylation of Smad 2/3; 4) downregulation of Smad7; 5) increased secretion of TNF-α and IL-1β; 6) downregulation of miR-431-5p, and overexpression of miR-21 and miR-106b. Two regulatory mechanisms via TGF-β1/Smad pathway affect ferroptosis: A) induction of ferroptosis by GPX4 suppression; B) upregulation of Bcl-2 inhibits ferroptosis. The NF-κB signaling pathway is activated through the following mechanisms: 7) upregulation of CagA; 8) *H. pylori* infection; 9) increased production of IL-1β and TNF-α; 10) downregulation of p53; 11) overexpression of IKK2. NF-κB pathway suppresses ferroptosis through two mechanisms: C) reduction in ROS levels; D) upregulation of GPX4 and Nrf2

The TGF-β1/Smad signaling pathway plays a dual role in the development and invasion of GC [60]. It inhibits tumor growth in the early stages of GC but promotes the invasion and metastasis of cancer cells in the later stages [61]. This pathway can be activated in GC cells by multiple factors (Fig. 4).

Overexpression of TGF-β1 ligands in GC cells could continuously activate the TGF-β/Smad signaling pathway through autocrine or paracrine pathways [62] (circle marked 1 in Fig. 4). In addition, TGF-β1 activates downstream signals by binding to its receptors, and overexpression of TGF-β1 type 1 and 2 receptors could enhance or sustain the activation of the TGF-β1/Smad signaling pathway [63, 64] (circle marked 2 in Fig. 4). High levels of phosphorylated Smad 2/3 could also lead to persistent activation of TGF-β1 signaling in GC cells via formation of the Smad2/3-Smad4 complex [65] (circle marked 3 in Fig. 4). On the other hand, Smad7 suppresses the TGF-β1/Smad pathway through negative feedback [66], and its downregulation or inactivation in GC cells has been shown to activate this pathway [67] (circle marked 4 in Fig. 4). Furthermore, increased secretion of TNF-α and IL-1β in GC tissues promotes TGF-β1/Smad signaling by regulating rhomboid 5 homolog 2 (RHBDF2) [68] (circle marked 5 in Fig. 4). Several microRNAs (miRNAs) have also been identified that target TGF-β1 receptors or Smad proteins. For example, downregulation of miR-431-5p in GC cells stimulates TGF-β1/Smad signaling by activating zinc finger E‐box binding homeobox 1 (ZEB1) [69] (circle marked 6 in Fig. 4). In contrast, overexpression of miR-21 and miR-106b in GC cells could activate the TGF-β1/Smad pathway by downregulating PTEN and Smad7 [70, 71] (circle marked 6 in Fig. 4).

Previous studies showed that TGF-β1 could induce redox imbalance and enhance lipid peroxidation by inhibiting GPX4 and increasing ROS levels, which can lead to ferroptosis in osteoarthritis and hepatocellular carcinoma, respectively [72, 73] (circle marked A in Fig. 4). However, TGF-β1 might also promote cell survival and inhibit ferroptosis by upregulating Bcl-2 in liver and brain damage [74] (circle marked B in Fig. 4). This suggests that the role of TGF-β1/Smad in ferroptosis is highly context-dependent, influenced by factors such as cell type, disease stage, and crosstalk with other signaling pathways. Although there is currently no direct evidence that the TGF-β1/Smad signaling pathway affects ferroptosis in GC, a previous study showed that TGF-β1 acted as a major driver of epithelial-mesenchymal transition and fibrosis in advanced GC. These processes created a state of high oxidative stress, which synergized with TGF-β1's ability to promote lipid peroxidation [75]. Consequently, GPX4 activity might be inhibited, leading to the induction of ferroptosis. Therefore, we hypothesize that the pro-ferroptotic effect of TGF-β1 might be the more prevalent outcome in late stage of GC contexts. The anti-ferroptotic effect of TGF-β1 via Bcl-2 might represent a compensatory survival mechanism in a subset of cells or under specific conditions. The role of the TGF-β1/Smad pathway in ferroptosis is more complex and needs further investigation.

In summary, the TGF-β1/Smad pathway has demonstrated effects on promoting ferroptosis in GC. Although there is no clinical trial to induce ferroptosis by targeting TGF-β1/Smad pathway for GC therapy, activation of TGF-β1-induced ferroptosis could attenuate the development of hepatoma [73]. Therefore, whether activating the TGF-β/Smad pathway can alleviate GC progression by inducing ferroptosis remains to be validated.

### NF-κB signaling pathway

*Helicobacter pylori* can activate the NF-κB pathway in host cells by injecting the oncogenic protein CagA [76], which promotes degradation of IκB through IκB kinase (IKK), and facilitates the release of p65/p50 NF-κB subunits [77] (circle marked 7 in Fig. 4).

Furthermore, *H. pylori* can also activate MyD88 and IRAK in GC cells upon binding to Toll-like receptor 4 (TLR4), resulting in NF-κB activation [78] (circle marked 8 in Fig. 4). GC cells produce high levels of pro-inflammatory cytokines like, such as IL-1β and TNF-α, which can also activate the NF-κB pathway. For instance, IL-1β activates the MyD88 dependent pathway by binding to IL-1 receptors, which promotes IKK-mediated degradation of IκB and subsequent nuclear translocation of NF-κB [79] (circle marked 9 in Fig. 4). In addition, TNF-α binds to its receptor and recruits TRADD and RIP1, and the resulting complex leads to IκB degradation and NF-κB release [80]. The inactivation of p53 in cancer cells maintains the NF-κB signaling pathway in a continuously activated state [81], and indirectly activates the pathway by increasing the expression of pro-inflammatory factors, such as IL-6 and TNF-α [82, 83] (circle marked 10 in Fig. 4). Overexpression of IκB kinase 2 (IKK2), the primary catalytic subunit of the IKK complex, promotes tumor growth through NF-κB activation [84] (circle marked 11 in Fig. 4). On the other hand, IκB binds to and inhibits the nuclear translocation of NF-κB. Therefore, accelerated degradation of IκBα or mutations in its encoding gene might lead to sustained activation of NF-κB pathway [85].

Various chemotherapeutic drugs trigger ferroptosis in GC cells by inhibiting NF-κB [86, 87]. Studies reported that NF-κB inhibition in HepG2 cells could increase the levels of ROS, MDA, and Fe^2+^, and concurrently deplete GSH, thereby intensifying lipid peroxidation and inducing ferroptosis [88] (circle marked C in Fig. 4). In contrast, other studies showed that NF-κB could induce ferroptosis by suppressing antioxidant genes, such as *GPX4 and Nrf2*, in liver and brain tissue [89, 90]. This apparent contradiction could be explained by the differential roles of NF-κB, affecting the expression of numerous ferroptosis-related genes [91]. For instance, activating the NF-κB signaling pathway could promote SOD2 and GPX4 expression in GC tissue [92] (circle marked D in Fig. 4), however, another study demonstrated that NF-κB signaling pathway activation could reduce the expression of the crucial ferroptosis factors such as heme oxygenase 1 (HMOX1) and GPX4, which ultimately promoted oxidative stress and ferroptosis in cerebrovascular disease [93]. Therefore, the specific role of the NF-κB signaling pathway in ferroptosis shows strong organ-specific heterogeneity. In GC cells or tissue, the effects might be more inclined to inhibit ferroptosis.

Therefore, inhibiting the NF-κB signaling pathway might also be a therapeutic strategy to overcome treatment resistance via inducing ferroptosis.

### Hippo signaling pathway

**Fig. 5:**
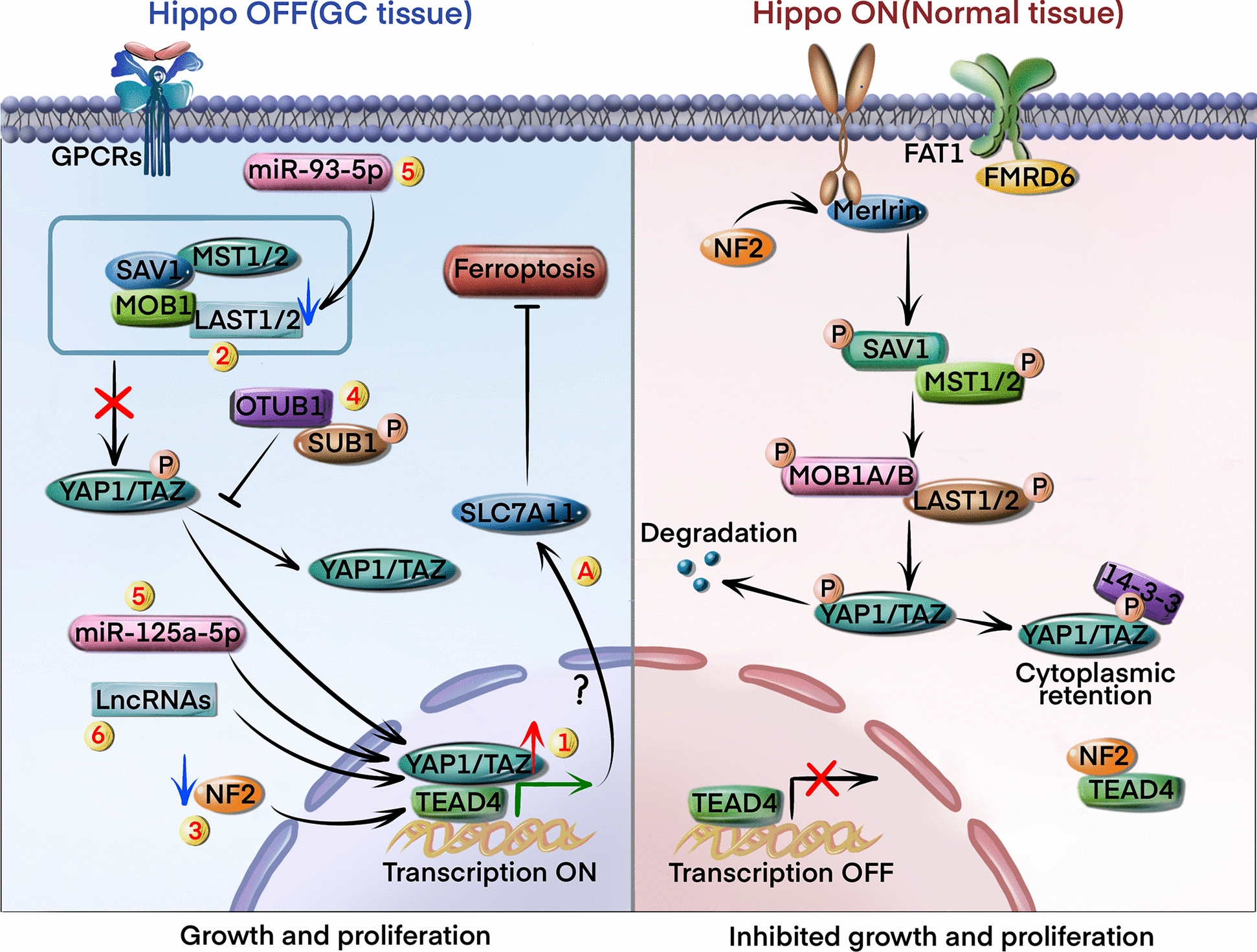
Hippo signaling pathway regulates ferroptosis. In normal tissues, MST1/2 activity is regulated in a phosphorylation-dependent manner by upstream factors such as FAT1 and Merlin. MST1/2 promotes LATS1/2-mediated phosphorylation of YAP/TAZ and prevents its nuclear translocation. Phosphorylated YAP/TAZ undergoes proteasomal degradation in the cytoplasm (Hippo on, right panel). Numerous pathways trigger the dephosphorylation cascade of MST1/2-LATS1/2-YAP/TAZ, resulting in the nuclear translocation of YAP/TAZ: 1) increased localization of YAP/TAZ in the nucleus; 2) downregulation of the LATS1/2 gene promotes nuclear translocation of YAP; 3) upregulation of NF2; 4) upregulation of OTUB1 and DAB2 inhibits YAP/TAZ degradation; 5) upregulation of some miRNAs inhibits YAP1 and TAZ degradation; 6) upregulation of specific lncRNAs downregulates YAP1. The role of Hippo signaling upregulated GPX4 to inhibit ferroptosis in GC cells(Hippo off, left panel)

The Hippo signaling pathway is a highly conserved pathway that includes mammalian STE20-like protein kinase 1/2 (MST1/2) and large tumor suppressor 1/2 (LATS1/2). Inhibition of Hippo signaling promotes the proliferation and migration of GC cells via dephosphorylation of the downstream Yes-associated protein (YAP) and transcriptional co-activator with PDZ-binding motif (TAZ) [94, 95]. Numerous mechanisms are responsible for Hippo suppression (Fig. 5).

Overexpression or nuclear localization of YAP/TAZ suppresses the Hippo signaling pathway in GC cells [96] (circle marked 1 in Fig. 5). In addition, downregulation of the *LATS1/2* gene could directly promote nuclear translocation of YAP [97] (circle marked 2 in Fig. 5). The tumor suppressor gene neurofibromatosis 2 (NF2) encodes the cytoskeletal protein merlin, and is frequently mutated in GC [98]. NF2 inhibits TEA domain transcription factor 4 (TEAD4) palmitoylation and induces the cytoplasmic translocation of TEAD4 [99] (circle marked 3 in Fig. 5). Furthermore, some deubiquitinases, including OTU deubiquitinase ubiquitin aldehyde binding 1 (OTUB1) and disabled-2 (DAB2), could deubiquitinate YAP1 and TAZ, respectively, at several lysine sites, and inhibit their degradation [100, 101] (circle marked 4 in Fig. 5). Several miRNAs have been identified that promote GC growth by targeting the Hippo pathway. For example, miR-125a-5p could promote the nuclear translocation of TAZ and transcriptionally activate its target genes [102] (circle marked 5 in Fig. 5). In addition, miR-93-5p could inactivate the hippo signaling pathway by inhibiting the expression of LATS2 [103] (circle marked 5 in Fig. 5). Overexpression of certain long non-coding RNAs (lncRNAs), such as SNHG1, 00662, FER1L4, and 00649, in GC tissues could inactivate the Hippo pathway by downregulating YAP1 [104–107] (circle marked 6 in Fig. 5).

There is some new evidence that the Hippo pathway regulates ferroptosis through various effectors. A recent study showed that pharmacologically inducing the tyrosine phosphorylation of YAP/TAZ in GC could increase iron influx, elevate lipid peroxidation, and heighten sensitivity to ferroptosis [108] (circle marked A in Fig. 5). Meanwhile, exosomal SLC1A5 could drive GC progression to enhance the nuclear translocation of YAP1 and subsequent upregulation of GPX4, resulting in ferroptosis suppression [109]. This suggests that activation of the Hippo signaling pathway might be a promising avenue for future research to identify novel therapeutic targets for GC via inducing ferroptosis.

## Clinical evaluation of TCM for GC treatment

TCM formulations, including herbal formulae and patent drugs, have been used as complementary therapies for GC for decades [110]. The following sections summarize published randomized controlled trials (RCTs) on TCM for GC treatment. Although the underlying mechanisms of these formulations are not yet fully classified, these studies provide scientific evidence to support the application and potential of TCM as an auxiliary treatment for GC.

### Herbal formulae

The modified Banxiaxiexin Decoction (MBXXXD), comprising ten herbs (Table 1), is a classic prescription that has been used to treat gastrointestinal diseases for thousands of years. MBXXXD could significantly prolong the survival of patients with GC and improve their clinical symptoms and quality of life scores [111] (Table 2).

**Table 1:** Species of source the prescribed formulae traditional Chinese medicine for GC treatment in clinical trials

Name	Species, source
Modified Banxiaxiexin decoction (MBXXXD)	*Pinellia ternata* (Thunb.) Makino (9 g), *Coptis chinensis* Franch. (3 g), *Scutellaria baicalensis* Georgi (15 g), *Zingiber officinale* Roscoe (9 g), *Panax ginseng* C.A.Mey. (9 g), *Ziziphus jujuba* Mill. (9 g), *Glycyrrhiza glabra* L. (9 g), *Atractylodes macrocephala* Koidz. (15 g), *Iphigenia indica* (L.) A.Gray ex Kunth (15 g), *Radix actinidiae* Chinensis (15 g)
Fuzheng Hewei decoction (FZHWD)	*Codonopsis pilosula* (Franch.) Nannf. (15 g), *Astragalus membranaceus* Bunge (30 g), *Ophiopogon japonicus* (Thunb.) Ker Gawl. (10 g) *Panax quinquefolius* L. (6 g), *Atractylodes macrocephala* Koidz. (10 g), *Poria cocos* (Schw.) Wolf (10 g), Pinellia ternata (Thunb.) Breit. (10 g), *Citrus reticulata* Blanco (6 g), *Angelica sinensis* (Oliv.) Diels (10 g), *Coix lacryma-jobi* L. var. ma-yuen (Roman.) Stapf. (10 g), *Akebia quinata* (Houtt.) Decne. (15 g), *Actinidia arguta* (Sieb. & Zucc.) Planch. ex Miq. (15 g), *Bletilla striata* (Thunb.) Rchb.f. (10 g), *Citrus aurantium* L. (6 g), *Curcuma aromatica* Salisb. (10 g), *Bambusa tuldoides* Munro (10 g), *Coptis chinensis* Franch. (6 g). *Typha angustifolia* L. (10 g), The dried feces of T*rogopterus xanthipes* Milne-Edwards (10 g)
Wei Chang’An	*Pseudostellaria heterophylla* (Miq.) Pax (12 g), *Atractylodes lancea* (Thunb.) DC. (12 g), *Poria cocos (Schw.)* Wolf (30 g), *Sargentodoxa cuneata* (Oliv.) Rehder & E.H.Wilson (30 g), *Concha ostreae* (30 g), *Prunella vulgaris* L. (9 g)
Herbal formula for invigorating spleen	*Atractylodes macrocephala* Koidz. (12 g), *Poria cocos* (Schw.) Wolf (30 g), *Sargentodoxa cuneata* (Oliv.) Rehder & E.H.Wilson (30 g), *Ostrea gigas* Thunberg (30 g), *Prunella vulgaris* L. (9 g)
Modified Taohongsiwu decoction	*Prunus persica* (L.) Batsch (30 g), *Carthamus tinctorius* L. (30 g), *Rehmannia glutinosa* (Gaertn.) DC. (30 g,), *Angelica sinensis* (Oliv.) Diels (30 g), *Conioselinum anthriscoides* 'Chuanxiong'(15 g), *Paeonia lactiflora* Pall. (15 g), *Neolitsea cassia* (L.) Kosterm. (15 g), *Cyathula officinalis* K.C.Kuan (15 g), *Glycyrrhiza glabra* L. (6 g), and *Ziziphus jujuba* Mill. (three pieces)
Liujunzi decoction (Rikkunshito)	*Codonopsis pilosula* (Franch.) Nannf. (9 g), *Atractylodes macrocephala* Koidz. (9 g), *Poria cocos* (Schw.) Wolf (9 g), *Glycyrrhiza glabra* L. (4.5 g), *Citrus reticulata* Blanco (3 g), *Pinellia ternata* (Thunb.) Makino (6 g)
Weidiao-3 (WD-3)	*Codonopsis pilosula* (Franch.) Nannf. (10 g), *Atractylodes macrocephala* Koidz. (10 g), *Pinellia ternata* (Thunb.) Makino (6 g), *Poria cocos* (Schw.) Wolf (10 g), *Citrus reticulata* Blanco (6 g), Coix lacryma-jobi var. ma-yuen (Rom.Caill.) Stapf (30 g), *Dioscorea oppositifolia* L. (15 g), *Hordeum vulgare* L. (15 g), *Eriobotrya japonica* (Thunb.) Lindl. (15 g), Glycyrrhiza glabra L. (3 g)
Fuzheng Qingdu Decoction (FZQDD)	*Astragalus membranaceus* (Fisch.) Bunge (30 g)*, Atractylodes macrocephala* Koidz. (10 g)*, Poria cocos (Schw.)* Wolf (10 g), *Dioscorea polystachya* Turcz. (15 g)*, Coix lacryma-jobi* var. ma-yuen (Rom.Caill.) Stapf (20 g)*, Citrus reticulata* Blanco Pericarpium (10 g), *Cynanchum otophyllum* C.K.Schneid. (10 g)*, Smilax china* L. (30 g), *Salvia chinensis* Benth. (20 g)*, Solanum lyratum* Thunb. (30 g), *Scleromitrion diffusum* (Willd.) R.J.Wang (30 g), *Perilla frutescens* (L.) Britt. (10 g), *Agastache rugosa* (Fisch. et Mey.) O.Ktze. (10 g), *Solanum nigrum* L. (30 g), *Pinellia ternata (Thunb.)* Ten. ex Breitenb. (10 g), *and Glycyrrhiza uralensis* (Fisch.) (3 g)
Sijunzi decoction (SJZD)	*Codonopsis pilosula* (Franch.) Nannf. (20 g), *Atractylodes macrocephala* Koidz. (20 g), *Poria cocos* (Schw.) Wolf (20 g), *Glycyrrhiza uralensis* Fisch. ex-DC. (10 g)
YiQi QingDu decoction	*Pseudostellaria heterophylla* (Miq.) Pax (15 g). *Atractylodes macrocephala* Koidz. (12 g), *Poria cocos* (Schw.) Wolf (12 g), *Scleromitrion diffusum* (Willd.) R.J.Wang (30 g), *Glycyrrhiza uralensis* Fisch. ex DC. (6 g), *Scutellaria barbata* D.Don (30 g), *Actinidia arguta* (Siebold & Zucc.) Planch. ex Miq.(20 g), *Gynostemma pentaphyllum* (Thunb.) Makino (20 g), *Astragalus membranaceus* (Fisch.) Bunge (30 g), *Citrus reticulata* Blanco (10 g)
Jianpi Huoxue Jiedu prescription	*Astragalus mongholicus* Bunge (30 g), *Ligustrum lucidum* W.T.Aiton (20 g), *Coix lacryma-jobi* L.var.mayuen(Roman.) Stap (30 g), *Polyporus umbellatus* (Pers.) Fr. (15 g), *Agrimonia eupatoria* L. (30 g), *Spatholobus suberectus* Dunn (30 g), *Sophora flavescens* Aiton (15 g), *Scleromitrion diffusum* (Willd.) R.J.Wang (30 g), *Clematis chinensis* Osbeck (30 g), *Scrophularia ningpoensis* Hemsl. (20 g)
Wenyang Yiqi Decoction	*Codonopsis pilosula* (Franch.) Nannf. (30 g), *Atractylodes macrocephala* Koidz. (20 g), *Astragalus mongholicus* Bunge (30 g), *Cinnamomum verum* J.Presl (6 g), *Bupleurum scorzonerifolium* Willd. (6 g), *Magnolia officinalis* Rehder & E.H.Wilson (15 g), *Citrus reticulata* Blanco (10 g), *Aconitum carmichaelii* Debeaux (12 g), *Zingiber officinale* Roscoe (10 g), *Coptis chinensis* Franch. (3 g)
Fuzheng Yiliu Granule (FZYLG)	*Hedysarum polybotrys* Hand.-Mazz. (30 g), *Angelica sinensis* (Oliv.) Diels (10 g), Curcuma phaeocaulis Valeton (10 g), *Patrinia intermedia* (Hornem.) Roem. & Schult. (30 g)
Jinlongshe Granule (JLSG)	*Arisaema erubescens* (Wall.) Schott (15 g), *Pinellia ternata* (Thunb.) Makino (15 g), *Cremastra appendiculata* (D.Don) Makino (15 g), *Paris yunnanensis* Franch. (30 g)
Shenbai Granules (SBG)	*Sophora flavescens* Aiton (4.5 g), *Scleromitrion diffusum* (Willd.) R.J.Wang (10 g), *Codonopsis pilosula* (Franch.) Nannf. (7.5 g), *Atractylodes macrocephala* Koidz. (6 g), *Coix lacryma-jobi* var. ma-yuen (Rom.Caill.) Stapf (10 g), *Coptis chinensis* Franch. (1.5 g), *Prunus mume* (Siebold) Siebold & Zucc (4.5 g). *Zingiber officinale* Roscoe (3 g)
Yiqi Bushen Koufuye (YQBSK)	*Astragalus mongholicus* Bunge (20 g), *Poria cocos* (Schw.) Wolf (12 g), *Ligustrum lucidum* W.T.Aiton (12 g), *Lycium barbarum* L. (12 g), *Curcuma longa* L. (12 g)*, Scutellaria barbata D.Don(10 g), Actinidia arguta* (Siebold & Zucc.) Planch. ex Miq. (30 g), Coix lacryma-jobi L. (30 g), *Akebia trifoliata* (Thunb.) Koidz. (30 g), *Ziziphus jujuba* Mill. (30 g)*, Glycyrrhiza glabra* L. (6 g)
Yiwei Xiaoyu granules (YWXY)	*Talinum paniculatum* (Jacq.) Gaertn., *Panax notoginseng* (Burkill) F.H.Chen, *Atractylodes macrocephala* Koidz., *Coix lacryma-jobi* var. ma-yuen (Rom.Caill.) Stapf, *Angelica apaensis* R.H.Shan & C.C.Yuan, *Aesculus chinensis* Bunge, *Fritillaria thunbergii* Miq

**Table 2:** Summary of the prescribed formulae and natural products from traditional Chinese medicine for GC treatment in clinical trials

Name	Research design	Treatment method (number)		Treatment Duration	Outcome	Reference
		Treatment group	Control group			
Modified Banxiaxiexin decoction (MBXXXD)	RCT	MBXXXD + chemotherapy (n = 73; 8.4 g per packet; two packs each time; twice a day)	Placebo + chemotherapy (n = 73)	18 weeks	Prolonged survival and improved clinical symptoms	[111]
Fuzheng Hewei decoction (FZHWD)	RCT	Fuzheng Hewei decoction + chemotherapy (1 dose/day, n = 34)	Placebo + chemotherapy (n = 32)	12 weeks	Increased survival rate and reduced some adverse reactions	[112]
Wei Chang’An	RCT	Chemotherapy + Wei Chang’An (one dosage/day, n = 145)	Chemotherapy (n = 254)	3 months	Prolonged survival	[113]
Herbal formula for invigorating spleen	RCT	Chemotherapy + Herbal formula for invigorating spleen (one dosage/day, n = 89)	Chemotherapy (n = 139)	6 months	Improved the prognosis of elderly patients	[114]
Modified Taohongsiwu decoction	RCT	Modified Taohongsiwu decoction (once a day, for 30 min each time; n = 60)	Pyridoxine (100 mg, twice daily, orally; n = 32)	3 months	Decreased Hand-foot syndrome and improved patients' quality of life	[115]
Liujunzi decoction (Rikkunshito)	RCT	Liujunzi decoction (7.5 g/day, n = 4)	Control group (n = 7)	4 weeks	Improved gastric emptying and ameliorated postoperative symptoms	[116]
Weidiao-3 (WD-3)	RCT	Weidiao-3 + Immunotherapy (1 dose/day, n = 25)	Immunotherapy (n = 26)	6 weeks	Improved the quality of life and the efficacy of immunotherapy	[117]
Fuzheng Qingdu Decoction (FZQDD)	RCT	Chemotherapy + FZQDD (one dosage/day, n = 29)	Chemotherapy (n = 33)	2 weeks	Improved the cancer-related symptoms and the quality of life	[118]
Sijunzi decoction (SJZD)	RCT	Sijunzi decoction (100 ml/day, n = 21)	Control group (n = 20)	9 days	Increase T-cell subsets, serum albumin (ALB), prealbumin (PA) and transferrin (TRF)	[122]
YiQi QingDu decoction	RCT	Chemotherapy + YiQi QingDu decoction (1 dose/day, n = 34)	Chemotherapy (n = 34)	6 months	Enhanced the life quality of patients and prolonged survival	[124]
Jianpi Huoxue Jiedu prescription	RCT	Chemotherapy + Jianpi Huoxue Jiedu prescription (1 dosage/day, n = 42)	Chemotherapy (n = 33)	12 months	Improved the quality of life and the immunity function	[125]
Wenyang Yiqi Decoction (WYYQD)	RCT	Wenyang Yiqi Decoction (1 dosage/day, n = 24)	Placebo (n = 24)	1 week	promoted the recovery of intestinal function	[126]
Fuzheng Yiliu Granule (FZYLG)	RCT	Fuzheng Yiliu Granule (9 g for each time, twice times per day, n = 43)	Control (n = 34)	15 days	Increased the apoptosis rate	[127]
Jinlongshe Granule (JLSG)	RCT	JLSG (one bag daily, n = 25)	Placebo (n = 25)	6 months	Improve the quality of life	[130]
Shenbai Granules (SBG)	RCT	SBG (twice a day, n = 167)	Placebo (n = 169)	3 months	Reduced the recurrence of adenoma	[131]
Yiqi Bushen Koufuye (YQBSK)	RCT	Chemotherapy + Yiqi Bushen Koufuye (twice daily, n = 28)	Chemotherapy (n = 19)	12 months	Increased the surviving rate and improved life quality	[132]
Yiwei Xiaoyu granules (YWXY)	RCT	YWXY (15 g/day, n = 36)	Weifuchun tablet (n = 36)	24 weeks	Relieved symptoms of atrophic gastritis	[133]

The Fuzheng Hewei Decoction (FZHWD) consists of 19 herbs (Table 1). The combination of chemotherapy and FZHWD extended the one-year survival of patients with GC compared to chemotherapy alone [112] (Table 2). Furthermore, FZHWD reduced the incidence of adverse reactions, such as leucopenia, nausea, vomiting, mucosal reaction, and fatigue [112].

Wei Chang’An is a traditional herbal formula (Table 1) prescribed for invigorating the spleen. Patients with GC treated with Wei Chang’An showed prolonged survival and fewer adverse events during a 3-year follow-up compared to the placebo group [113] (Table 2). Another herbal formula for spleen invigoration also improved the prognosis of elderly patients with GC without significantly impacting the survival duration [114] (Table 2), thereby warranting further investigation.

Certain chemotherapy drugs can induce hand-foot syndrome (HFS), a skin reaction that mainly appears on the palms and/or soles. The modified Taohongsiwu Decoction mitigated the HFS-induced pain in patients with GC (Table 1), and the HFS incidence rate was much lower in patients soaking with modified Taohongsiwu Decoction compared to those receiving pyridoxine [115] (Table 2).

Liujunzi Decoction, also known as Rikkunshito in Japan (Table 1), is routinely prescribed for invigorating the spleen. Rikkunshito improved gastric emptying rate and ameliorated postoperative symptoms in patients who underwent a pylorus-pre serving gastrectomy [116] (Table 2). Weidiao-3 (WD-3) is a modified version of Liujunzi Decoction that significantly improved the quality of life of patients with GC by relieving symptoms of dry mouth, altered taste, and reflux (Table 1). WD-3 also increased the relative abundance of *Bifidobacteria* and *Coprococcus* as well as the contents of isobutyric and isovaleric acid in the gut microflora of patients with GC, which was associated with improved outcomes of immunotherapy [117] (Table 2).

Fuzheng Qingdu Decoction (FZQDD) is composed of 16 herbs (Table 1). The combination of FZQDD and chemotherapy significantly improved the cancer-related symptoms and the quality of life of patients with GC compared to those receiving chemotherapy. In addition, FZQDD also increased the parasympathetic activity and decreased the sympathetic tone in patients with GC [118] (Table 2).

In addition to improving clinical symptoms, some TCMs also have beneficial effects on the immune function and nutritional status of cancer patients. Previous studies have shown a close crosstalk between the immune microenvironment and ferroptosis sensitivity, where immune activation could promote ferroptosis within cancer cells[119–121]. For instance, activated CD^8+^ T cells secrete IFN-γ which induces ferroptosis in cancer cells [120], while enhancing immune checkpoint blockade can suppress ferroptosis by activating the Nrf2/GPX4 system and reducing the amount of phospholipid PUFAs [121].

A single-blinded, controlled clinical trial demonstrated that Sijunzi Decoction (SJZD), a classical Chinese herbal formula consisting of four herbal components (Table 1), increased the proportion of T cell subsets, and the serum levels of albumin (ALB), prealbumin (PA) and transferrin (TRF) in patients with GC after surgery [122] (Table 2). Another RCT showed that SJZD attenuated gastritis and intestinal metaplasia of gastric mucosa, indicating its potential as a new drug for the treatment of pre-cancerous lesions and GC prevention [123]. In addition, YiQi QingDu Decoction combined with chemotherapy improved the therapeutic effects and mitigated the clinical symptoms of patients with GC, accompanied by an increase in T lymphocyte subsets, and a decrease in CEA and CA19-9 levels [124] (Tables 1 and 2).

Several herbal medicines have also been shown to inhibit postoperative GC metastasis. For instance, the Jianpi Huoxue Jiedu prescription consisting of ten herbs (Table 1), showed apparent anti-metastasis effects in patients with GC when combined with chemotherapy. It also improved the quality of life of the patients with GC by lowering whole blood viscosity and enhancing immune function [125] (Table 2). Wenyang Yiqi Decoction (Table 1) facilitated the recovery of intestinal function in patients with GC after radical gastrectomy [126] (Table 2).

### Patent Chinese drugs

Fuzheng Yiliu Granule (FZYLG), which consists of four herbal medicines (Table 1), could increase apoptosis of tumor cells in patients with GC by upregulating NF-κB [127] (Table 2).

Shenqi Fuzheng Injection (SQFZJ) is derived from *Codonopsis pilosula* (Franch.) Nannf. and *Astragalus mongholicus* Bunge. The combination of SQFZJ and chemotherapy led to tumor remission and disease stabilization in patients with GC, which was attributed to the increased activity of NK cells, macrophages, and T lymphocyte subgroups [128]. Another study showed that SQFZJ effectively improved the clinical symptoms in patients with GC and alleviated chemotherapy-induced adverse reactions [129].

Jinlongshe Granule (JLSG) is an orally administered recipe composed of four TCMs (Table 1). A double-blind, placebo-controlled RCT demonstrated that JLSG improved the quality of life of patients with GC according to the European Organization for Research and Treatment of Cancer Core Quality of Life Questionnaire C30 (EORTC QLQ-C30) [130] (Table 2).

Shenbai Granule (SBG) consists of eight medicinal plant materials (Table 1). SBG significantly reduced the recurrence of adenomas and sessile serrated lesions in patients with GC over a 2-year follow-up, although the underlying mechanism was not elucidated [131] (Table 2).

Yiqi Bushen Koufuye (YQBSK) is an oral formulation extracted from 11 herbs (Table 1). The combination of YQBSK and chemotherapy could prevent postoperative metastasis of stomach cancer, increase survival rates, and improve the quality of life and immunological functions in patients with GC [132] (Table 2).

Yiwei Xiaoyu Granule (YWXY) is a composite preparation extracted from seven herbs (Table 1). A double-blind RCT showed that YWXY improved the Operative Link on GastricIntestinal Metaplasia Assessment (OLGIM) stage, relieved symptoms of atrophic gastritis induced by *H. pylori* infection (a risk factor for GC), and improved serum gastric function in patients with GC [133] (Table 2).

## Mechanisms underlying the therapeutic action of herbal medicines against GC

**Fig. 6:**
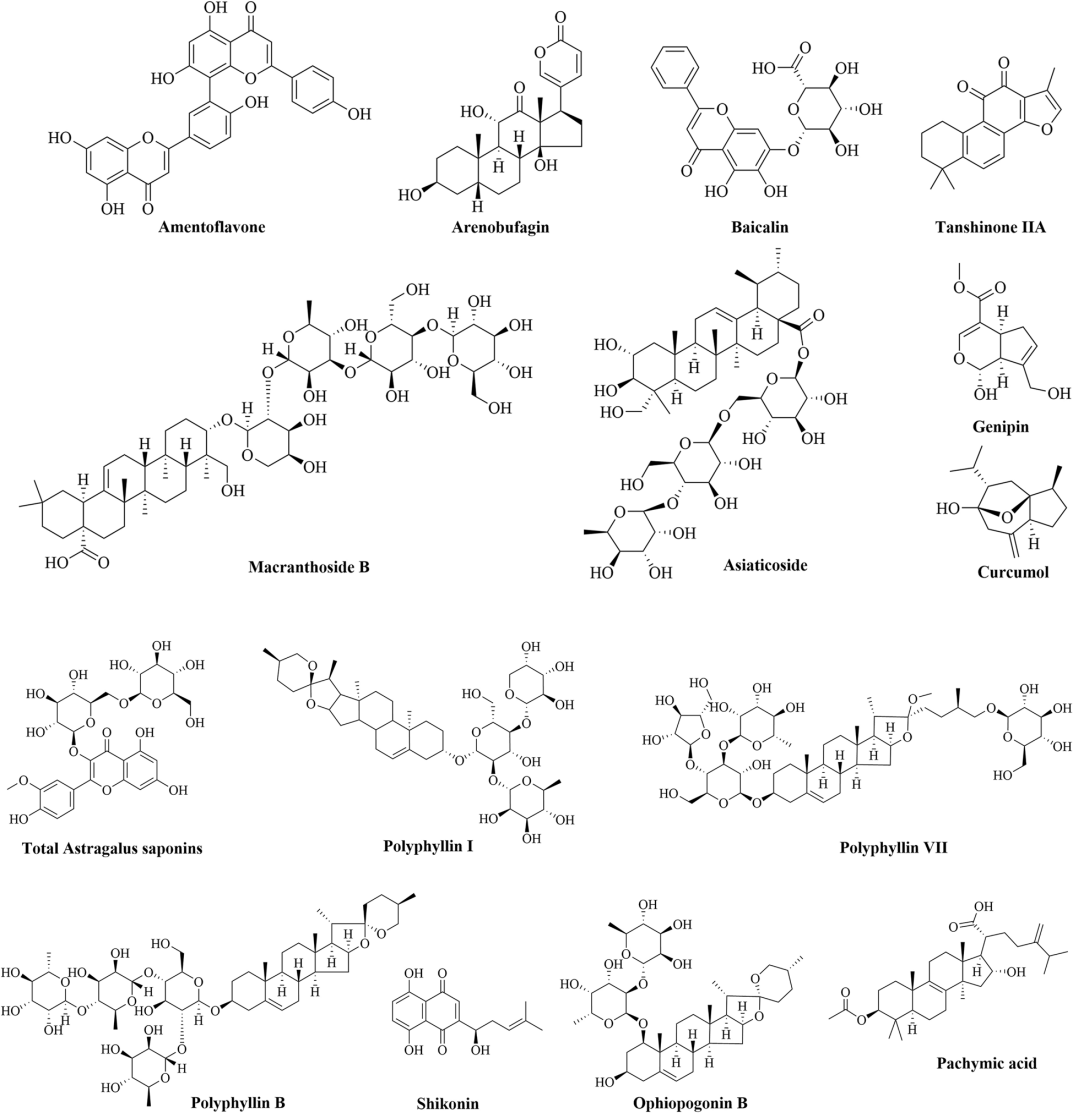
Chemical structures of representative bioactive compounds extreacted from Chinese medicine related to induce ferroptosis in GC

Studies on animal models have shown that some TCMs and their bioactive compounds can trigger ferroptosis and inhibit GC progression (Table 3 and Fig. 6).

**Table 3:** The mechanisms by which some chemical substances and herbs medicine ameliorate the progression of GC by triggering ferroptosis in animal models

Medicine form	Name	Type of Study	Treatment method		Treatment Duration	Outcomes	Targets or pathways	Year	References
			Experiment group	Control group					
Chinese medicine	YJD	MKN-45/DDP GC cell lines and its xenograft tumor mode	YJD (11.7, 23.4 g/kg/day)	Saline (i.g.)	24 h/21 days	Suppressed the xenograft growth via inducing ferroptosis	Nrf2/GPX4↓,ROS↑	2024	[52]
	JPYZXZ	HGC-27 and MKN-45 cell lines; tumor xenograft with injecting MKN-45 cell	JPYZXZ (2, 4, 8 mg/ml in cell; 15, 30, 45 mg/kg in mice)	Saline	24 h/14 days	Inhibited the proliferation and migration of GC tumor	GPX4/xCT↓	2025	[134]
	JYQHD	GES-1, BGC-823, SGC-901 and MKN-45 cell line; tumor xenograft with injecting MKN-45 or SGC-7901	JYQHD-medicated serum and JYQHD injection (39 g/kg/day in nude mice)	Normal serum	24 h/19 days	Suppressed the activity of GC cells via inducing ferroptosis	COL12A1↓	2023	[135]
	FZNZ	GES-1 and MC cells	FZNZ (4000 μg/ml)	PBS	24 h	Decreased MC cells viability and induced ferroptosis	GPX4/GSH↓	2022	[136]
	QZJWD	HGC-27 and AGS cells; tumor xenograft with injecting MKN-45 or SGC-7901	QZJWD (0.5, 1, and 2 mg/mL in cell; 1,2,4 g/mL in mice)	Saline	24 h/28 days	Suppressed the proliferation and migration of GC cells	GPX4↓	2025	[137]
	YQHY	AGS GC cell line	YQHY (11.20 mg/ml)	PBS	24 h	Inhibited AGS growth and induce ferroptosis	JAK2-STATs/ACSL4↑	2022	[139]
Single herbs	ACP	HGC-27 GC cell line and its zebrafish xenograft	APC (90,180,360 mg/ml)	PBS injection	24 h/2 days	Suppressed the xenograft growth via inducing ferroptosis	GPX4/SLC7A11/ Fer-1↓	2020	[140]
	EM	MKN45/DDP cells and tumor xenograft with injecting MKN45/DDP cells	EM(0.125, 0.25, 0.5 μg/mL in cell; 2 g/kg/day in nude mice)	Saline	224 h/8 days	Inhibited the migration and invasive ability of GC cells and induced ferroptosis	NF-κB/Snail↓, PI3K/AKT/mTOR↓	2024	[86]
Nature products	Amentoflavone	AGS and HGC-27 GC cell line and tumor xenograft injecting AGS cells	Amentoflavone (5, 10, 20 or 40 μM in cell; 80 mg/kg in nude mice)	PBS	24 h/28 days	Suppressed GC proliferation and induced ferroptotic cell death	miR-496/ATF2 axis↑	2023	[141]
	ArBu	MGC-803 GC cell line and tumor xenograft injecting MGC-803cells	ArBu (0.25, 0.5, 1, 2, 4, 8 μM in cell; 1, 3, 5 mg/kg in nude mice)	Saline	24 h/28 days	Inhibited GC cell proliferation and triggered ferroptosis	Nrf2/SLC7A11/GPX4↓		[142]
	Asiaticoside	AGS and HGC27 GC cell lines and tumor xenograft with injecting AGS cells	Asiaticoside (1, 2 and 4 μM in cell and 50 mg/kg in nude mice)	Vehicle	24 h/36 days	Reduced tumor volume and induced ferroptosis	GPX4/SLC7A11/IFN-γ↓	2024	[143]
	Baicalin	HGC-27 GC cell line	Baicalin (50, 100, 200, 400 μM)	Vehicle	24 h	Induced GC cells ferroptosis	SLC7A11/GPX4↓, ROS↑	2024	[144]
	CUR	MKN-45, AGS cell and tumor xenograft with injecting AGS cell	CUR (10, 25, 50, 100, 125, 150 μM; 30 mg/kg in nude mice)	Saline	24 h and 21 days	Inhibited the GC tumors growth and induced ferroptosis	GPX4/KEAP1/Nrf2 ↓	2024	[145]
	Genipin	AGS, HGC-27, MKN-45 and BGC-823 GC cell lines	Genipin (25, 50, 75, 100, 125 µM in cell)	DMSO	24 h	Inhibits cell viability and colony formation	GPX4 and SLC7A11↓	2024	[146]
	MB	AEG cell	MB (9.5, 12.7 µM in cell lines)	DMSO	25 h	Inhibited AEG cell growth and induced ferroptosis	Nrf2↓	2025	[147]
	Tan IIA	BGC-823 and NCI-H87 GC cell lines and tumor xenograft with injecting BGC-823 cells	Tan IIA (2,4 μM in cell; 50 mg/kg in nude mice)	Saline	24 h/21 days	Induced ferroptosis via increasing lipid peroxidation	p53/ Fer-1↓	2020	[148]
		SGC-7901 and BGC-823 GC cell lines	Tan IIA (25,50, 125, 250 μM)	PBS	24 h	Reduce gastric cancer cell stemness	SLC7A11/Fer-1↓	2022	[149]
	TAS	SGC-7901 GC cell line	TAS (0, 50, 100, and 200 μg/mL)		48 h	Inhibited SGC-7901 proliferation	SLC7A11/GPX4↓	2025	[150]
	Polyphyllin I	AGS and MKN-45 GC cell lines and tumor xenograft injecting AGS and MKN-45 cells	Polyphyllin I (1, 2, 4 μM in cell; 3 mg/kg/day in nude mice)	Saline	24 h/15 days	Inhibited the GC growth	Nrf2/FTH1↓	2023	[152]
		AGS, MKN-45 GC cell lines and tumor xenograft	Polyphyllin I (1, 2, 4 μM in cell; 3 mg/kg/day in nude mice)	Saline	24 h/15 days	Inhibited the GC growth	miR-124-3p↑, NF2 ↓	2023	[153]
	Polyphyllin B	NUGC-3, MKN-1, MKN-45, HGC-27, and NUGC-4 cell line; and tumor xenograft injecting MKN-1 cells	Polyphyllin B (0.5, 1.0 or 2.0 µM in cell; 2.5, 5 mg/kg in nude mice)	Saline	24 h/21 days	Inhibited tumor growth and induced ferroptosis	GPX4/NOCA4↓	2023	[154]
	Polyphyllin VII	AGS and NCI-N87 GC cell lines and tumor xenograft injecting BGC823 cells	Polyphyllin VII (1.2,1.4, 1.6 μM in cell; 2.0 mg/kg in nude mice)	Saline	24 h/30 days	Inhibited tumor growth and induced ferroptosis	FTH1↓	2023	[155]
	RGP	AGS GC cell line and tumor xenograft injecting AGS cells	RCP (50, 100, and 200 μg/mL in cell; 75, 150, 300 mg/kg in nude mice)	Saline	24 h/ 37 days	Inhibited the proliferation of AGS cells and induced ferroptosis	PI3K/AKT↓	2024	[157]
	PA	SGC-7901, AGS cell lines and tumor xenograft injecting SGC-7901 cells	PA(10, 20, 40 μM in cells; 100, 200, 400 mg/kg in nude mice)	Saline	48 h/14 days	Inhibited the proliferation of AGS cells and induced ferroptosis	GPX4/SLC7A11↓	2024	[156]
	Ophiopogonin B	AGS and NCI‑N87 GC cell lines and tumor xenograft injecting AGS cells	Ophiopogonin B (10, 20 μM in cell; 50 mg/kg in nude mice)	Saline	24 h/14 days	Reduced tumor volume and induced ferroptosis	GPX4/xCT↓	2022	[158]
	Shikonin	AGS, HGC-27, MKN-45 and BGC-823 GC cell line	Shikonin (0, 1, or 1.5 μM)	PBS	24 h	Inhibited the cell proliferation and induced ferroptosis	GPX4/ferritin↓	2024	[159]

*ACP* Actinidia chinensis Planch, *ArBu* Arenobufagin, *CUR* Curcumol, *DDP* Cisplatin, *EM* Eremias multiocellata, *Fer-1* Ferrostatin-1, *FTH1* ferritin heavy chain 1, *FZNZ* Fuzheng Nizeng Decoction, *GPX4* glutathione peroxidase 4, *JPYZXZ* Jianpi Yangzheng Xiaozheng granule, *JYQHD* Jian Yun Qing Hua Decoction, *MB* Macranthoside, *QZJWD* Qizhu Jianwei decoction, *RGP* Red ginseng polysaccharide, *SLC7A11* solute carrier family 7 member 11, *Tan IIA* Tanshinone IIA, *TAS* Total Astragalus saponins, *ULK1* Unc-51-like autophagy-activating kinase 1, *xCT* cystine-glutamate antiporter, *YJD* Yi-qi-hua-yu-jie-du decoction, *YQHY* Yiqi Huayu Decoction. ↑ for upregulation, ↓ for downregulation

### Herbal formulae

The Yi-qi-hua-yu-jie-du Decoction (YJD) enhanced the survival of patients with GC by inhibiting the phosphorylation cascade in the AKT/GSK3β pathway and downregulating Nrf2 expression [52].

Jianpi Yangzheng Xiaozheng Granule (JPYZXZ) is composed of twelve different types of TCMs [134]. A study demonstrated that JPYZXZ could inhibit the proliferation and migration of GC cells. This effect is associated with downregulating the expression of GPX4 and xCT [134].

Jian Yun Qing Hua Decoction (JYQHD) inhibited the growth and sphere-forming ability of GC cells by inducing ferroptosis through the downregulation of COL12A1 [135].

Fuzheng Nizeng Decoction (FZNZ) was effective against gastric precancerous lesions (GPL), thereby reducing the incidence of GC. Furthermore, FZNZ decreased the viability of GC cells and induced ferroptosis by downregulating GPX4/GSH [136].

As a conventional TCM, Qizhu Jianwei Decoction (QZJWD) has been used to treat GC; however, the underlying mechanisms are not fully elucidated. A study revealed that QZJWD treatment could suppress the proliferation and migration of GC cells, which was associated with reducing GPX4 [137].

Network pharmacology is an emerging field that integrates systems biology, network analysis, and pharmacology to explore the complexity of drug action. It is particularly suitable for predicting TCM targets due to their complexity [138]. Using the network pharmacology approach, a study demonstrated that 205 active compounds in Yiqi Huayu Decoction (YQHY) could target ferroptosis [139]. Furthermore, YQHY freeze-dried powder inhibited the growth of the human gastric adenocarcinoma cell line AGS and induced ferroptosis by activating the Janus kinase 2 (JAK2) -signal transducer and activator of transcription 3 (STAT3) pathway and upregulating acyl-CoA synthetase long-chain family member 4 (ACSL4) [139].

### Single herbs

*Actinidia chinensis* Planch (ACP) or Chinese kiwifruit significantly inhibited the proliferation and migration of HGC-27 cells and increased the accumulation of ROS by inhibiting GPX4 and SLC7A11. Furthermore, administration of ACP suppressed the xenograft growth by inhibiting Ferrostatin-1 (Fer-1) [140].

*Eremias multiocellata* (EM) is a TCM with anti-cancer effects. It inhibited the migration and invasive ability of MKN45/DDP cells and increased ROS levels and lipid peroxidation to induce ferroptosis by regulating the NF-κB/Snail and PI3K/AKT/mTOR signaling pathways [86].

### Bioactive compounds

Amentoflavone is a natural multifunctional biflavonoid that could suppress the proliferation of GC cells and induce ferroptosis via the miR-496/ATF2 axis [141].

Arenobufagin (ArBu) is a natural monomer extracted from the secretion of the Chinese toad. A recent study showed that ArBu could inhibit the proliferation of MGC-803 cells and was linked to ferroptosis. Further investigation revealed that it could reduce the expression of Nrf2, SLC7A11, and GPX4 [142].

Asiaticoside, a triterpenoid derivative extracted from *Centella asiatica* (L.) Urb, increased the levels of Fe^2+^ ions and ROS in GC cells and downregulated GPX4 and SLC7A11, resulting in decreased GSH levels. Furthermore, asiaticoside treatment reduced the size and weight of tumors in a mouse model and downregulated GPX4, SLC7A11, IFN-γ, and the Wnt/β-catenin pathway [143].

Baicalin is an effective component of *Scutellaria baicalensis*. A recent study showed that baicalin disrupted iron homeostasis and inhibited antioxidant mechanisms in GC cells, resulting in iron accumulation and lipid peroxide aggregation. At the molecular level, baicalin triggered ferroptosis by upregulating the tumor suppressor gene *p53*, and activating the SLC7A11/GPX4/ROS pathway [144].

Curcumol (CUR) is a sesquiterpene compound found in some TCMs, such as zedoary and turmeric. The combination of CUR and cisplatin could inhibit the growth of subcutaneous GC tumors and induce ferroptosis via downregulating GPX4 and P62/KEAP1/Nrf2 signaling pathways [145].

Genipin is an iridoid constituent in Gardeniae Fructus. A network pharmacological analysis identified that lipid- and ROS-related pathways involved in ferroptosis ranked among the common genipin-GC targets. Further investigation showed that Genipin treatment decreased levels of GPX4 and SLC7A11, induced the accumulation of lipid peroxidation intracellularly, thereby leading to ferroptosis in GC cells [146].

Macranthoside B (MB) is a saponin compound extracted from honeysuckle. It could inhibit AEG cell growth by regulating iron homeostasis and trigger ferroptosis by inhibiting the expression of Nrf2 [147].

Tanshinone IIA (Tan IIA) is a pharmacologically active component isolated from the rhizome of *Salvia miltiorrhiza* Bunge. Our previous study showed that Tan IIA could induce ferroptosis in the BGC-823 and NCI-H87 cell lines by increasing lipid peroxidation and upregulating Ptgs2 and Chac1 [148]. In addition, another study showed that Tan IIA induced lipid peroxidation and ferroptosis in SGC-7901 and BGC-823 cells by upregulating SLC7A11 and suppressing Fer-1 [149].

Total Astragalus saponins (TAS) is a natural product derived from *Astragalus membranaceus*. TAS could induce ferroptosis of SGC-7901 cells by promoting the expression of SIRT3 and ACSL4 and inhibiting the expression of SLC7A11 and GPX4 [150].

Polyphyllin I is a natural anti-tumor compound extracted from *Paris polyphylla* [151]. A study showed that polyphyllin I inhibited GC growth by increasing the intracellular ROS/lipid peroxides, through downregulating Nrf2 and ferritin heavy chain 1 (FTH1) [152]. Furthermore, polyphyllin I increased miR-124-3p expression in GC cells and promoted ferroptosis by downregulating Nrf2 expression [153]. Polyphyllin B is another dioscin isolated from *Paris polyphylla* Hayata plants. It could increase the level of Fe^2+^ by transporting Fe^3+^ into the cell by TFR1. In addition, it could suppress tumor growth in an orthotopic mouse model of GC by regulating the expression of GPX4 and NOCA4 [154]. Polyphyllin VII is another component extracted from *P. polyphylla*, which induced autophagy-mediated ferroptosis in GC cells by decreasing FTH1 and activating the Unc-51-like autophagy-activating kinase 1 (ULK1) [155].

Pachymic acid (PA) is a natural triterpenoid extracted from *Poria cocos* (Schw. Wolf) could induce ferroptosis via the PI3K/AKT signalling pathway which could suppress the expression of GPX4 and SLC7A11[156].

Red ginseng polysaccharide (RGP) is an active component of red ginseng. RGP effectively inhibited the proliferation of AGS cells and induced ferroptosis by inhibiting the expression of PI3K/AKT and aquaporin 3 (AQP3) [157].

Ophiopogonin B is extracted from Radix *Ophiopogon japonicus* and has been shown to reduce tumor volume and induce ferroptosis by blocking the GPX4/xCT system [158].

Shikonin is an active ingredient extracted from the roots of *Lithospermum erythrorhizon*. A study found that shikonin could decrease the expression of GPX4 by suppressing its synthesis and decreasing ferritin levels in GC cells [159].

## Discussion

The low efficacy of GC treatment is mainly attributed to the development of resistance to chemotherapy and radiotherapy, as well as to the limitations of targeted therapies [160]. Although the underlying mechanism is unclear, ferroptosis resistance is a key factor influencing the chemoresistance, proliferation and metastasis of GC cells [161]. This review demonstrates that multiple signaling pathways are involved in the regulation of ferroptosis in GC cells, including the Wnt/β-catenin, PI3K/AKT/mTOR, TGF-β1/Smad, NF-κB and Hippo pathways. Although distinct molecular mechanisms trigger these pathways, the activated pathways act synergistically and often regulate each other. For instance, β-catenin can regulate the PI3K/AKT signaling pathway [162], and TGF‑β1 can activate both the PI3K/Akt and Wnt/β-catenin pathways [163, 164]. Therefore, inhibiting a single pathway cannot optimize ferroptosis levels, and drugs targeting individual molecules may not be effective in reversing treatment resistance through ferroptosis. This provides a theoretical basis for using TCM as a complementary therapy to alleviate drug resistance.

This review summarized the results of 17 RCTs using TCM, including 12 herbal formulae and five patent Chinese drugs (Table 1 and Table 2), as complementary therapies against GC. Five RCTs demonstrated that TCM combined with chemotherapy could prolong the survival of patients with GC or increase their chances of survival [111–113, 124, 132]. Other studies demonstrated that TCM could reduce some adverse reactions and improve the quality of life of patients (Table 2). *Poria cocos* (Schw.) Wolf and *Codonopsis pilosula* (Franch.) Nannf. are present in different medicinal formulae and patent Chinese drugs and have shown therapeutic effects against GC. However, the underlying molecular mechanisms have not been investigated in the RCTs, and the role of ferroptosis has also not been ascertained yet. A recent bioinformatics study demonstrated that almost ten ferroptosis-related genes were differentially expressed in GC tissues, and some herbal medicines, such as *Salvia miltiorrhiza* Bunge, *Coptis chinensis* Franch., and *Reynoutria japonica* Houtt. may target these genes in GC cells [165]. Meanwhile, no natural compounds extracted from TCM have been used for GC therapy in clinical studies.

This review also summarized 25 preclinical studies demonstrating that Chinese herbal medicines and their bioactive compounds could ameliorate GC progression by triggering ferroptosis (Table 3). Furthermore, six herbal formulae have been shown to induce ferroptosis by decreasing the expression of certain antioxidant genes and promoting oxidative stress, although the upstream signaling pathways remain unknown. Similarly, ten monomers extracted from these herbs could induce ferroptosis by targeting the Wnt/β-catenin pathway [143] and PI3K/AKT pathway [157]. Most natural products extracted from herbs can also suppress some antioxidant genes, such as *Nrf2* and *GPX4* (Table 3). However, the marked structural heterogeneity observed among these pharmacological agents (Fig. 6) suggests that their suppressive effects on antioxidant gene expression may occur through divergent molecular pathways, necessitating comprehensive mechanistic studies for full elucidation. These preclinical studies have been published after 2020, indicating a surging interest in using TCM formulations to alleviate drug resistance in patients with GC by inducing ferroptosis.

Despite the compelling preclinical evidence that various TCM formulae and compounds can induce ferroptosis in GC models, their path to clinical application is fraught with significant challenges. First, the bioavailability of numerous bioactive TCM compounds is often low, hampered by poor aqueous solubility and rapid systemic metabolism and elimination [166, 167]. Second, herbal formulas may contain toxic compounds, such as heavy metals, pesticide residues, and biotoxin which are hardly to remove[168]. Third, quality control and standardization present a major hurdle, since the chemical composition of herbal extracts can vary drastically based on the plant's geographic origin, harvest time, and processing methods. Although some methods, such as metabolomics-based screening or chromatographic fingerprinting, can be used to ensure batch-to-batch consistency for natural medicinal substances [169, 170], the process remains complex and costly. This makes it difficult to apply in clinical trials where reproducible efficacy is required. Finally, bridging the gap from preclinical findings to human trials requires careful consideration, since animal models or monocultures cannot fully recapitulate the human tumor microenvironment and immune context.

Furthermore, the toxic effects of ferroptosis are also critical aspects for evaluating its clinical translational potential. Studies have shown that ferroptosis could induce seizure severity, anxiety-like behaviors [171], inflammation, and mucus secretion [172]. Therefore, although TCM offers a rich resource of ferroptosis inducers, a thorough and cautious evaluation of their safety is an indispensable step before they can be widely adopted in clinical oncology practice.

Future efforts must focus on developing standardized clinical-grade Chinese medicine products, and employing advanced drug delivery systems to improve bioavailability. In addition, innovative clinical trials incorporating imaging and molecular biomarkers are essential to directly confirm the safety of ferroptosis induction in human patients and to correlate it with therapeutic efficacy.

## Conclusion and perspectives

The limitations of this review should be noted. First, the medicinal formulations discussed in this review were assessed in only one RCT, which might raise questions regarding the accuracy of the results. Second, the sample sizes of these RCTs were small and were single-center studies; most of the studies were published in Chinese. These factors inevitably increase the susceptibility to participant selection bias and limit the generalizability of the results. Third, the lack of long-term follow-up data precludes any definitive conclusions regarding overall survival benefit and long-term safety profiles. Furthermore, the studies using animal models did not include chemotherapy or combination therapy groups as controls, which is inconsistent with the actual situation in clinical practice. Although there are numerous shortcomings in the published studies, they do provide benchmarks for future research. These findings also highlight the need for future large-scale, multi-center, phase III RCTs with extended follow-up periods to conclusively determine efficacy and integrate these novel approaches into clinical practice for GC.

## Data Availability

No datasets were generated or analysed during the current study.

## Abbreviations

ACSL4: Acyl-CoA synthetase long-chain family member 4
ALB: Albumin
ALOXs: Arachidonate lipoxygenases
APC: *Adenoma polyposis coli*
AQP3: Aquaporin 3
BAD: Bcl-2-associated death promoter
CTNNB1: Catenin beta 1
DAB2: Disabled-2
DKK1: Dickkopf-related protein 1
DKK4: Dickkopf-related protein 4
DMT1: Divalent metal transporter 1
EGFR: Epidermal growth factor receptor
EM: *Eremias multiocellata*
FPN: Ferroportin
FTH1: Ferritin heavy chain 1
FZHWD: Fuzheng Hewei decoction
FZNZ: Fuzheng Nizeng decoction
FZQDD: Fuzheng Qingdu decoction
FZYLG: Fuzheng Yiliu granule
GC: Gastric cancer
GPL: Gastric precancerous lesions
GPX4: Glutathione peroxidase 4
GSH: Glutathione
GSK-3β: Glycogen synthase kinase-3β
HER2: Human epidermal growth factor receptor-2
HFS: Hand-foot syndrome
HMOX1: Heme oxygenase 1
IKK: IκB kinase
IKK2: IκB kinase 2
JAK2: Janus kinase 2
JLSG: Jinlongshe Granule
JYQHD: Jian Yun Qing Hua decoction
LATS1/2: Large tumor suppressor ½
LOX: Lipoxygenase
MBXXXD: Modified banxiaxiexin decoction
MDA: Malondialdehyde
miRNAs: MicroRNAs
MST1/2: Mammalian STE20-like protein kinase ½
NF2: Neurofibromatosis 2
Nrf2: Nuclear factor-E2-related factor 2
OTUB1: OTU deubiquitinase ubiquitin aldehyde binding 1
PA: Prealbumin
PIK3CA: Phosphatidylinositol-4,5-bisphosphate 3-kinase alpha subunit
POR: Cytochrome P450 oxidoreductase
PTEN: Phosphatase and tensin homolog
PUFAs: Polyunsaturated fatty acids
RCTs: Randomized controlled trials
RGP: Red ginseng polysaccharide
RHBDF2: Rhomboid 5 homolog 2
Rheb: RAS homolog enriched in brain
ROS: Reactive oxygen species
SBG: Shenbai Granule
SCD1: Stearoyl-CoA desaturase 1
sFRP4: Secreted frizzled-related protein 4
SJZD: Sijunzi Decoction
SLC3A2: Solute carrier family 3 member 2
SLC7A11: Solute carrier family 7 member 11
SNHG11: Small nucleolar host gene 11
SQFZJ: Shenqi Fuzheng injection
SREBP1: Sterol regulatory element binding protein 1
STAT3: Signal transducer and activator of transcription 3
STEAP3: Six-transmembrane epithelial antigen of prostate 3
TAZ: Transcriptional co-activator with PDZ-binding motif
TCM: Traditional Chinese medicine
TEAD4: TEA domain transcription factor 4
TFR1: Transferrin receptor 1
TLR4: Toll-like receptor 4
TRF: Transferrin
ULK1: Unc-51-like autophagy-activating kinase 1
WD-3: Weidiao-3
xCT: Cystine-glutamate antiporter
YAP: Yes-associated protein
YJD: Yi-qi-hua-yu-jie-du Decoction
YQBSK: Yiqi Bushen Koufuye
YQHY: Yiqi Huayu Decoction
YWXY: Yiwei Xiaoyu Granule
ZEB1: Zinc finger E‐box binding homeobox 1
